# COVID-19: the use of immunotherapy in metastatic lung cancer

**DOI:** 10.2217/imt-2020-0096

**Published:** 2020-04-30

**Authors:** Alexander P Davis, Michael Boyer, Jenny H Lee, Steven C Kao

**Affiliations:** 1 Chris O'Brien Lifehouse, Department of Medical Oncology, 119–143 Missenden Road, Camperdown NSW 2050, Australia; 2 Sydney Medical School, University of Sydney, NSW 2006, Australia; 3 Faculty of Medicine & Health, Macquarie University, NSW 2109, Australia

**Keywords:** COVID-19, duration of therapy, immune checkpoint inhibitors, immunotherapy, lung cancer

Lung cancer remains among the most lethal malignancies with a high mortality rate, which is in part due to the metastatic nature at the time of diagnosis. Untreated, the prognosis of metastatic non-small-cell lung cancer (NSCLC) is poor with a median overall survival of 7 months [1]. The introduction of drugs that target the axis between programmed death-1 (PD-1) and its ligand (PD-L1) have rapidly changed the treatment and prognosis of NSCLC over the past decade. Use of immunotherapy as monotherapy for the second-line setting [2] or in the first-line setting for PD-L1-high tumors [3] has resulted in significant improvements in overall survival. Newer first-line protocols looking at combination immunotherapy with chemotherapy have seen benefits across the board, including both squamous and nonsquamous histology, EGFR mutant and ALK rearrangements [4–6]. The emergence of the novel coronavirus severe acute respiratory syndrome ([SARS]-CoV-2 or COVID-19) pandemic poses significant challenges for the treatment of all cancer patients, but in particular lung cancer patients where an increase in mortality has already been reported [7,8]. Here, we re-evaluate the risks, benefits and delivery of immunotherapy for NSCLC patients during the COVID-19 pandemic.

The clinical manifestations and severity of COVID-19 are broad but typically manifest as fever, cough, dyspnoea and myalgia [9]. Initial reports suggest that 81% of cases are mild and the remainder are classified as either severe or critical [10]. In this subset of patients, cough and fever can be present for approximately 7–10 days prior to the development of acute respiratory distress syndrome (ARDS), acute cardiac injury and acute kidney injury [11]. Patients with severe COVID-19 admitted to the intensive care unit were more likely to have proinflammatory cytokines such as IFN-γ, IP-10, MCP-1, IL-1β, IL-4 and IL-10 [9]. Initial pathological examination of ARDS in COVID-19 implies overactivation of T cells which may account for the severe immune mediated injury seen in patients [12]. While myocardial dysfunction and renal impairment are found in severe COVID-19, the cause and its relation to excessive inflammation is less clear. Currently, no vaccine or validated disease-modifying agents are available, however, treatment with high-dose glucocorticoids and medications that inhibit IL-6 such as tocilizumab have been reported [13]. No benefit of immunosuppression has yet been empirically demonstrated.

The immune-related adverse events (irAEs) of anti-PD-1 or anti-PD-L1 agents are typically inflammation caused by the immune system directed against organ-specific targets. While the exact pathophysiology of irAEs is not known, patients receiving anti-PD-1 or PD-L1 therapy can develop complications not limited to pneumonitis, myocarditis, nephritis, hepatitis, colitis, thyroiditis, hypophysitis, dermatitis, arthritis and encephalitis. No clear effect of microbial coinfection has been demonstrated in the type or frequency of irAEs. Specifically, there was no increased incidence of hepatitis in patients with chronic viral hepatitis nor increased risk of immune reconstitution in patients with HIV infection receiving treatment with immunotherapy [14,15]. However, there have been cases of PD-1 therapy causing a reactivation of latent tuberculosis through an increase in the immune response [16]. Case reports of fatal PD-1 induced encephalitis or myocarditis found Epstein Barr Virus positive lymphocytes in the affected histological region, suggesting some role of the infection in this idiosyncratic irAE [17]. Ultimately, the interaction between the immune system and microbes is complex and an area of emerging research. The impact of COVID-19 and whether it has any impact of irAE in lung cancer irAE will be an area of ongoing interest.

While PD-1 therapy was not in use during recent viral outbreaks, we can look at their impact on lung cancer patients to gain insight into the specific challenges that COVID-19 will bring. A total of 79 NSCLC were prospectively followed during the SARS outbreak in 2003 [18]. While there was considerable concern and anxiety regarding contracting SARS, there was minimal delay or interruption to treatment. The Middle East Respiratory Syndrome outbreak in 2015 demonstrated a mortality rate of 84% in cancer patients, which was twice as high when compared with nononcology patients [19]. Lung cancer patients comprised of 15.8% of these patients. The H1N1 influenza pandemic had considerably lower mortality but affected more people than the two previously mentioned coronavirus outbreaks. Hospital admission data during the H1N1 outbreak suggests that cancer within the last 12 months was one of the highest risk factors for death. During the H1N1 outbreak in 2009, hospital mortality for oncology patients admitted with H1N1 was up to 18.5% in some studies [20]. While the H1N1 outbreak was global and prolonged, it has not had the same societal impact as the COVID-19 outbreak, nor does it appear to be as lethal.

While the data for the impact of COVID-19 are limited and emerging, there are early indications that suggest significant impact on the oncology patient population, in particular, lung cancer. A retrospective case study across three hospitals in Wuhan identified 28 cancer patients suffering from COVID-19 [7]. Lung cancer was the most common cancer type in that group (25%) with one patient receiving immunotherapy. Lung cancer patients were more likely to have earlier symptoms of dyspnoea, to develop anoxia and more rapid progression of COVID-19 symptoms. Anticancer therapy administered within 14 days of presentation were more likely to have severe clinical outcomes such as admission to ICU, need for mechanical ventilation or death. A prospective cohort of laboratory confirmed COVID-19 cases in China identified 18 patients with a history of cancer [8]. Lung cancer was the most common cancer type (28%) with most patients being older (mean age 63.1 years) and in routine follow-up following treatment from cancer (75%). Patients with cancer were at higher risk of severe events compared with noncancer patients (39 vs 8%) and recent anticancer treatment was an independent risk factor for severe events. While evidence is limited currently, emerging reports suggest higher risk of adverse outcomes in cancer patients who received recent anticancer therapy. It is difficult to confirm that lung cancer patients were at higher risk based on this limited data without knowing the specific lung cancer incidence rate for this region of China. Nethertheless for lung cancer patients for whom treatment may be continued for up to 2 years, this may represent a significant risk.

Another challenge for lung cancer patients receiving immunotherapy and the current COVID-19 pandemic is a diagnostic one. The current WHO definition of a confirmed case of COVID-19 is based on laboratory confirmation typically via PCR. There is an evolving role for computed tomography imaging in the diagnosis of COVID-19, with advantages including rapid results and the ability to reflect the severity of ARDS [21]. There is a broad range of radiological features found in patients infected with COVID-19. Similarly, PD-1 inhibitor-induced pneumonitis can present with a broad range of radiological findings [22]. Most commonly, this presents as cryptogenic organizing pneumonia but in its more severe grade, is consistent with ARDS. Given the diagnostic overlap radiologically and the common clinical characteristics of cough and hypoxia, this can present a diagnostic challenge to the physician. The differential diagnosis of PD-1-induced pneumonitis may complicate the management of patients receiving PD-1 therapy and suspected of being infected with COVID-19.

Models of care associated with the delivery of immunotherapy in lung cancer will also have to be reconsidered. Currently, patients continue on immunotherapy for 2 years or longer in some instances should they continue to derive benefit. This can represent a significant period of time during which patients are attending clinic appointments, visiting pathology, radiology diagnostic centers and spending time in infusion centers. With no vaccine or disease modifying intervention currently available for COVID-19, the only strategy to reduce mortality associated with the outbreak is social distancing [23]. For patients stable on flat dosed immunotherapy, home-based treatment in selected patients may be utilized more frequently to minimize social interaction. Telehealth will undoubtedly replace a large proportion of face-to-face consultation and treatment protocols involving less chair time will likely be preferentially used.

The duration of treatment should also be considered when reducing direct patient interaction with the healthcare system. The duration of treatment for patients who are responding to immunotherapy has been a point of contention in lung cancer immunotherapy. Depending on the drug used and the line of therapy, some clinical trials have opted for 2 years of drug treatment or until progression. The Checkmate 153 study randomized patients who had completed 1 year of nivolumab to either treatment discontinuation with the option to resume nivolumab at time of progression and continuing nivolumab until progression. Importantly, this study reported superior progression free survival in patients who continued nivolumab [24]. 2 years of therapy has been standard in pembrolizumab-containing protocols. In patients who continued to respond after 2 years of therapy with pembrolizumab in the Keynote-10 trial, 75 of 79 patients had ongoing response [25]. With concerns about recent treatment for cancer increasing the risk of serious events with COVID-19, keeping the duration of treatment to an appropriate time would be important. Therefore, it may be reasonable to discuss stopping treatments in patients who have achieved a complete response or prolonged response for more than 2 years during this COVID-19 pandemic.

While recent cancer therapy appears to be a risk factor for serious events with COVID-19 infection, it is not clear whether the treatment modality mediates this risk. In a retrospective case series of cancer patients from three hospitals in Wuhan, six patients had received anticancer therapy in the preceding 14 days from COVID-19 diagnosis; two with cytotoxic chemotherapy, two with targeted therapy, one with radiotherapy and one with combination chemoimmunotherapy. Although it is difficult to draw a conclusion from this series due to small numbers, it is reasonable to make the assumption that cytotoxic chemotherapy would be more immunosuppressive than immunotherapy, and hence more harmful in patients with COVID19 coinfection. While there is a clear benefit of single-agent pembrolizumab versus conventional chemotherapy in the PD-L1 >50% group, there has been no comparison of single-agent immunotherapy versus chemoimmunotherapy in a clinical trial. Therefore, in the current climate, clinicians may be inclined to use protocols with single-agent immunotherapy versus combination chemoimmunotherapy, especially in patients with PD-L1 >50%. This has to be balanced with the inferior response rate seen in Keynote 024 of single-agent pembrolizumab (44%) versus combination chemoimmunotherapy (61.4% for PD-L1 >50%) in Keynote 189 [3,4].

The current end point to the COVID-19 pandemic likely involves reaching heard immunity or the mass availability of an effective vaccine. The time frame, delivery and toxicity of any potential vaccine is beyond the scope of this commentary. However, it is important to reiterate the safety of inactivated influenza vaccination for cancer patients receiving immunotherapy [26]. In the period preceding any potential COVID-19 vaccine, it is important that lung cancer patients continue to receive their yearly vaccinations and any potential exposure or hospitalization from seasonal influenza is avoided.

The impact of COVID-19 and the social distancing required to combat this pandemic has had a devastating impact on our society, healthcare and culture. We have highlighted the challenges within thoracic oncology, where significant number of patients whose life expectancy and quality of life have been vastly improved with novel anticancer therapy such as immunotherapy. Initial data from China’s experience with COVID-19 highlighting that these patients are at higher risk for serious events from COVID-19 should be taken into serious consideration when making decisions regarding patient selection for therapy, duration of therapy and the decision to combine immunotherapy with cytotoxic chemotherapy. Significant gains have been made in lung cancer morbidity and mortality since the introduction of PD-1 therapy. These benefits still exist in the ongoing COVID-19 pandemic, however, ongoing treatment may place a subset of patients at increased risk. As the COVID-19 crisis impacts upon healthcare systems across the world, we hope this commentary provides some guidance to thoracic oncologists as they deal with this emerging problem.
